# Hemagglutinin from the H5N1 Virus Activates Janus Kinase 3 to Dysregulate Innate Immunity

**DOI:** 10.1371/journal.pone.0031721

**Published:** 2012-02-16

**Authors:** Wei Xu, Minhui Chen, Nanhai Ge, Jun Xu

**Affiliations:** State Key Laboratory of Respiratory Disease, Guangzhou Institute of Respiratory Diseases, The First Affiliated Hospital of Guangzhou Medical University, Guangzhou Medical University, Guangzhou, People's Republic of China; University of Southern California, United States of America

## Abstract

Highly pathogenic avian influenza viruses (HPAIVs) cause severe disease in humans. There are no effective vaccines or antiviral therapies currently available to control fatal outbreaks due in part to the lack of understanding of virus-mediated immunopathology. In our study, we used hemagglutinin (HA) of H5N1 virus to investigate the related signaling pathways and their relationship to dysregulated innate immune reaction. We found the HA of H5N1 avian influenza triggered an abnormal innate immune signalling in the pulmonary epithelial cells, through an unusual process involving activation of Janus kinase 3 (JAK3) that is exclusively associated with γc chain and is essential for signaling via all γc cytokine receptors. By using a selective JAK3 inhibitor and JAK3 knockout mice, we have, for the first time, demonstrated the ability to target active JAK3 to counteract injury to the lungs and protect immunocytes from acute hypercytokinemia -induced destruction following the challenge of H5N1 HA *in vitro* and *in vivo*. On the basis of the present data, it appears that the efficacy of selective JAK3 inhibition is likely based on its ability to block multiple cytokines and protect against a superinflammatory response to pathogen-associated molecular patterns (PAMPs) attack. Our findings highlight the potential value of selective JAK3 inhibitor in treating the fatal immunopathology caused by H5N1 challenge.

## Introduction

Highly pathogenic avian influenza (HPAI) is an extremely contagious, multi-organ systemic disease [1]. One among multiple subtypes of influenza virus A, H5N1 viruses have caused 520 laboratory-confirmed infections in 15 countries, 307 of which were fatal, resulting in a fatality rate of approximately 60% since 2003 [2], which should be considered to have been a potentially serious pandemic threat [3]. Clinical observations indicated that acute lung injury and multiple organ dysfunction were the direct causes of death in H5N1-infected humans [4]–[5]. Laboratory findings revealed low peripheral blood T-lymphocyte counts and high chemokine and cytokine levels in H5N1-infected individuals, particularly in those who died. Levels of IP-10, MIG and MCP-1 (chemoattractants of monocytes and macrophages that are produced in bronchial epithelial cells and alveolar macrophages [6]–[8]) were elevated in patients with avian and human subtypes of influenza but were higher in H5N1-infected individuals and particularly high in those who died [6]. Levels of the neutrophil chemoattractant interleukin (IL)-8 were also elevated in H5N1-infected individuals, particularly in those who died [6]. The IL-8 chemokine is produced by bronchial epithelial cells and may function in the pathogenesis of acute respiratory distress syndrome (ARDS) [9], which may be particularly relevant to H5N1 influenza, as progression to respiratory failure is associated with the development of ARDS [10]–[11]. The clinical and pathological features in H5N1-infected humans and animal models suggest that high levels of viral replication combined with early robust host responses play a key role in pneumonia severity and outcome [6], [10]–[16].

The innate immune response of the cell is the first line of defence against viruses. Increasing evidence points to a key role of the innate immune system with its pattern recognition receptors (PRRs) in both infectious and non-infectious lung diseases, acute lung injury, pneumoconiosis and asthma [17]. Of PRRs, the well-known Toll-like receptors (TLRs) are expressed in alveolar macrophages, lung epithelial cells and in intraepithelial dendritic cells (DCs), which are either located at the cell surface or in endosomal membranes. These cells respond to infections by sensing pathogen-associated molecular patterns (PAMPs) and respond to endogenous molecules (danger-associated molecular patterns [DAMPs]) that are released after tissue damage [17]–[18]. The TLRs recruit different adapter molecules and initiate signalling pathways leading to the activation of NF-kB–dependent proinflammatory gene expression and/or to IRF3/7-mediated type I interferon (IFNa/b) expression [18]–[19]. An analysis of sections from the human respiratory tract demonstrated that H5N1 attached to the apical cell membrane of bronchiolar cells, type II pneumocytes and alveolar macrophages [20]. Therefore, activation of TLRs expressed in type II pneumocytes could help mediate the response to H5N1 viruses [21], contributing to the promotion of a destructive host immunity [22]–[24]. Although neuraminidase inhibitors are effective in treating avian influenza, especially if given within 48 h of infection, it is more difficult to prevent the resultant hypercytokinemia from developing if the patient does not seek timely medical assistance.

Corticosteroids have been used in some patients with HPAI H5N1, but no definitive role for steroids has been determined [25]–[26]. The evidence for corticosteroid use in other severe viral pneumonias, including varicella-zoster virus infection and severe acute respiratory syndrome (SARS), is also insufficient [7]–[8]. Several studies involving patients with sepsis and ARDS have suggested that high-dose corticosteroids actually increase the risk of secondary infections [27]. Thus, there is an urgent need to find ways of treating acute hypercytokinemia without compromising overall immunity. Because hemagglutinin, the major surface glycoprotein of H5N1, is responsible for viral binding to host receptors and initiating immediate signalling transduction upon viral invasion [28], the recombinant HA of H5N1 avian influenza virus (AIV) was used in the present study to investigate the signal transduction mechanisms for the dysregulated innate immune reaction. We have demonstrated that challenging respiratory epithelial cells with H5N1 HA exploited the JAK2/3/STAT1 and NF-κB signalling axis and resulted in a large release of cytokines, initiating a destructive innate immune response at early stages. Additionally, we found that a selective JAK3 inhibitor (JAK3inh) targeted to the key signal molecule in the inflammatory signal cascades has potential roles in the treatment of the inflammatory disorders, thereby protecting against a superinflammatory response to PAMPs attack.

## Results

### Morphological changes of the cultured pulmonary epithelial cells after exposure to the recombinant HA of AIV H5N1

**Figure 1:**
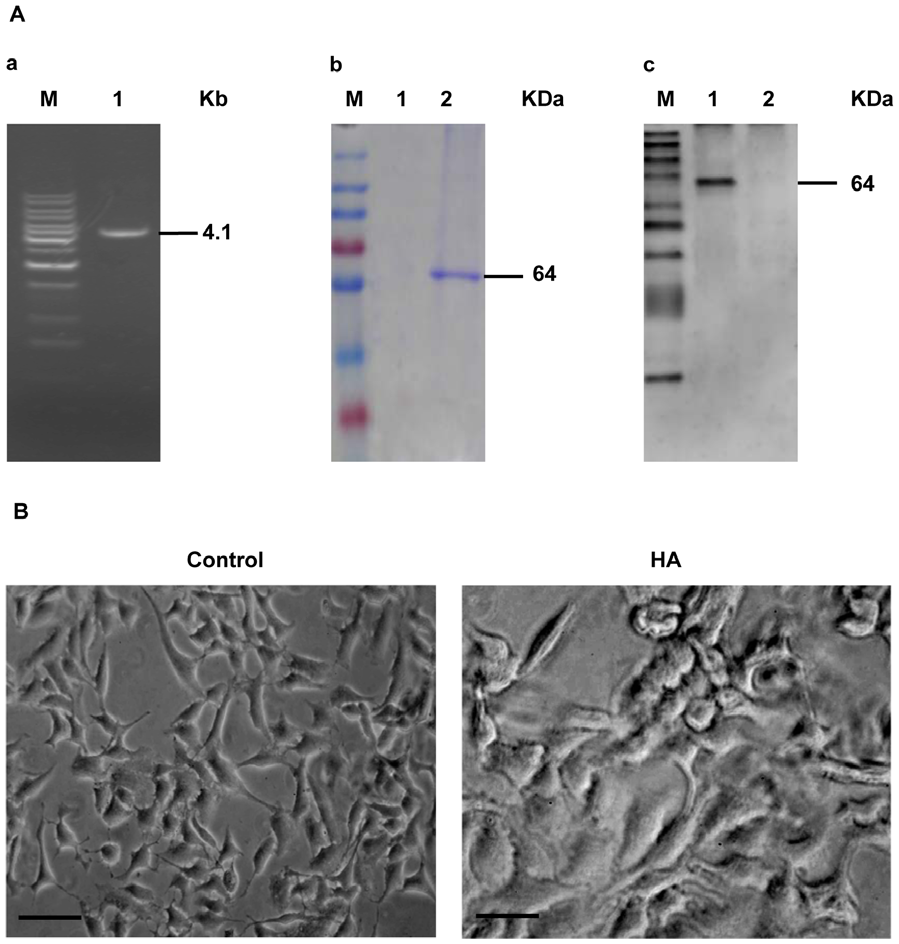
Evaluation of the expression and function of recombinant hemagglutinin protein (HA) of AIV H5N1. (A) Preparation of the recombinant HA protein. Identification of the Bacmid/HA recombinant (a) M, Marker, Lane 1, PCR product of Bacmid-HA. Recombinant HA purified from Bacmid/HA-transfected SF9 cells by Ni-NTA affinity chromatography (Coomassie Brilliant Blue staining) (b) M, prestained protein marker, Lane 1, Control (from SF9 cells transfected with blank bacmid), Lane 2, HA Purified from Bacmid/HA-transfected SF9 cells. Confirmation of HA recombinant by western blot analysis (c) M, Marker, Lane 1, HA Purified from Bacmid/HA-transfected SF9 cells, Lane 2, control. (B) Morphology changes in the recombinant HA-treated human pulmonary epithelial cells. A549 cells treated with 40 µg/ml HA (b) or the control (a) for 12 h (bar = 50 µm). The cells treated with HA become swollen, rounded and irregular in size and shape.

The cultured human pulmonary epithelial A549 cells were challenged with recombinant HA at 40 µg/ml. After 12 h of stimulation, the cells became swollen, rounded and irregular in size and shape with the appearance of intracellular vacuoles (**Figure 1Bb**) whereas the control cells did not (**Figure 1Ba**).

### Activation of JAK/STAT and NF-κB signalling in relation with innate immune inflammation in HA-challenged pulmonary epithelial cells

**Figure 2:**
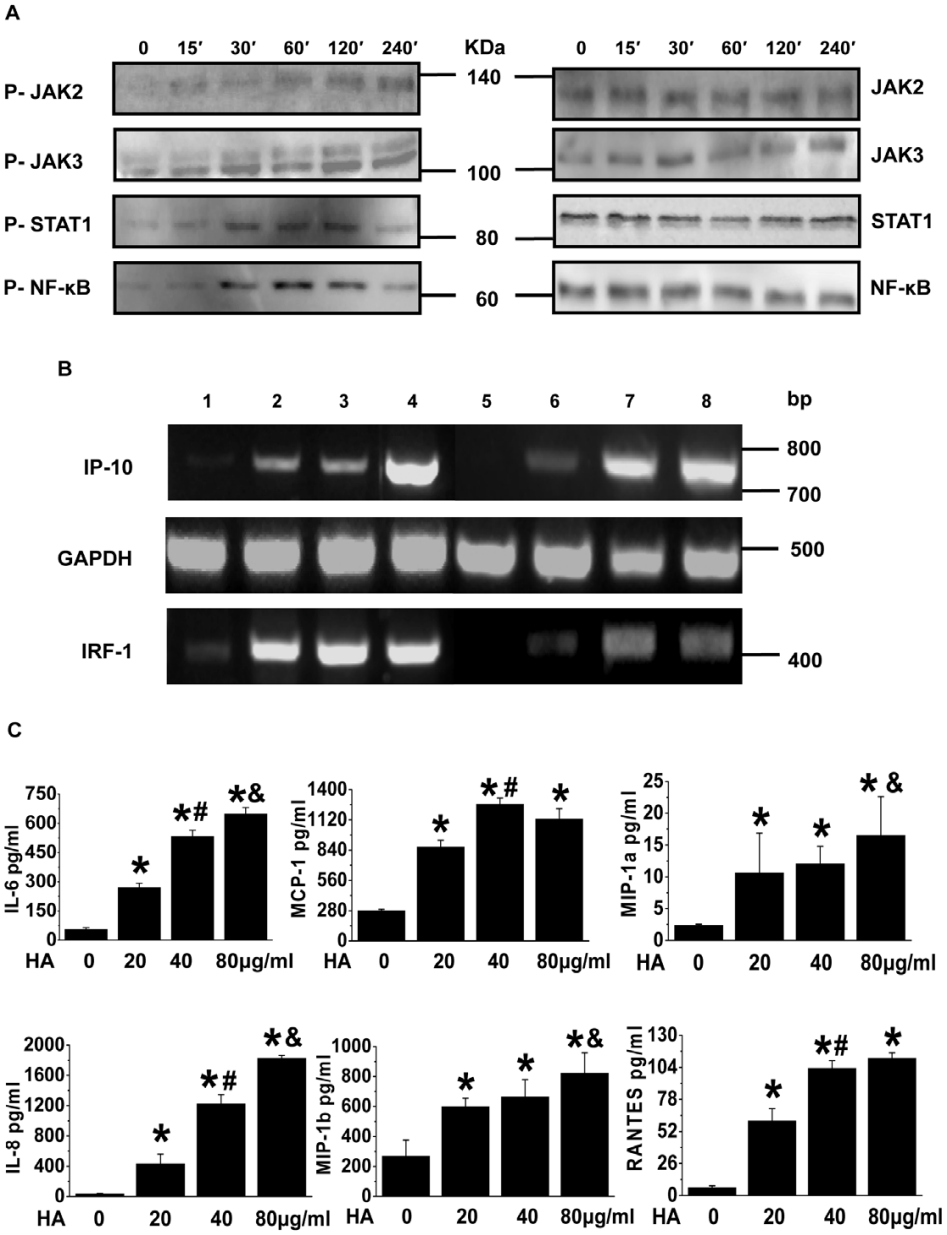
Impact of H5N1 HA on JAK/STAT and NF-κB signalling in the challenged pulmonary epithelial cells. (A) Detection of phosphorylated/nonphosphorylated JAK2, JAK3, STAT1 and NF-κB. Using specific antibodies, western blotting was performed in the A549 cells treated with the HA (40 µg/ml) for the indicated time periods. Representative blots from 3 replicates are shown. (B) The mRNA expression of IP-10 and IRF-1 on the HA-treated A549 cells. A549 cells were treated with the HA (40 µg/ml) for 1–4 h (Lane 1, 0 h; Lane 2, 1 h; Lane 3, 2 h; Lane 4, 4 h) or with the HA for 1 h at the indicated doses (Lane 5, control; Lane 6, 20 µg/ml; Lane 7, 40 µg/ml; Lane 8, 80 µg/ml) and then subjected to RT-PCR analysis for IP-10 and IRF-1. Representative gels from 3 replicates are shown. (C) Levels of IL-6, IL-8, MCP-1, MIP-1α, MIP-1β and RANTES in the supernatant of A549 cells treated with the indicated doses of HA for 12 h. *P<0.05 *vs.* control group; ^#^ *P*<0.05 *vs.* 20 µg/ml HA group; **&** *P*<0.05 *vs.* 40 µg/ml HA group.

We next tested if the recombinant HA could induce activation of JAK/STAT and NF-κB signal pathways, which are responsible for transcriptional activation of chemokines/cytokines genes and lead to an innate immune response against pathogens. We found that A549 cells exposed to HA have increasing levels of phosphorylation of JAK2, JAK3, STAT1 and NF-κB (**Figure 2A**), but not of JAK1 and STAT5 (data not shown), in a time-dependent manner.

Previous studies have demonstrated that phosphorylated STAT1 dimerises and translocates into the nucleus to activate the transcription of a number of genes, including IFN regulatory factor-1 (IRF-1). IRF-1 functions as a transcriptional factor for many antiviral genes, resulting in the production of chemokines (e.g., IP-10) that play critical roles in the infiltration of leukocytes into the site of inflammation [29]. Additionally, NF-κB dimers bind to κB sites within the promoter of the IP10 gene [30]. We therefore examined the transcription of IP-10 and IRF-1 genes in the HA-challenged A549 cells. As expected, our results showed an increased transcriptional induction of both genes following HA exposure (**Figure 2B**). Correspondingly, we detected a dose-dependent release of IL-6, IL-8, MCP-1, MIP-1α, MIP-1β and RANTES into the culture supernatants of the A549 cells 12 h after HA stimulation, as shown in **Figure 2C**.

### Effect of JAK3 activation on JAK/STAT and NF-κB signalling pathways in response to HA

**Figure 3:**
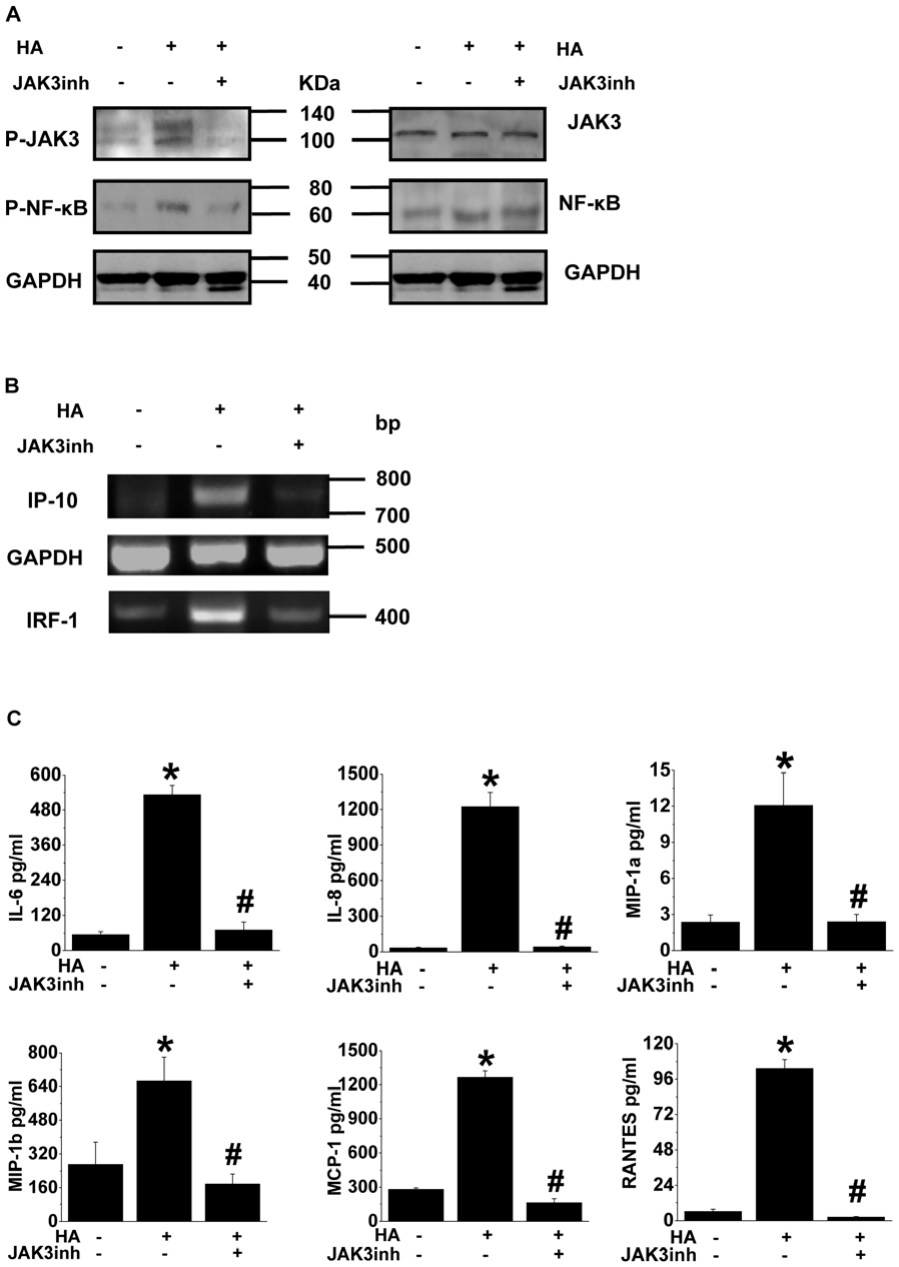
The role of JAK3 activation in JAK/STAT and NF-κB signalling upon challenge of HA. (A, B) Modulation of the phosphorylation of JAK3 and NF-κB and of the expression of the IP-10 and IRF-1 genes in the HA-treated A549 cells in the presence and absence of the JAK3 inhibitor VI. Western blot analysis (A) and RT-PCR (B) were performed to assess the signal pathways and the gene expression in the A549 cells challenged with HA (40 µg/ml) for 1 h in the absence and presence of the JAK3 inhibitor VI (760 nM). The measurement of the expression of GAPDH was performed simultaneously. Representative gels or blots from 3 replicates are shown. (C) Effects of treatment with the JAK3 inhibitor VI on the release of cytokines/chemokines from the HA-challenged A549 cells. A Liquidchip assay was performed on the supernatants of the A549 cells incubated with HA (40 µg/ml) for 12 h in the absence and presence of the JAK3 inhibitor VI (760 nM). *P<0.05 *vs.* control group; ^#^ *P*<0.05 *vs.* HA group.

Compared with the IFN-triggered JAK/STAT pathway, activation of JAK3 seems to be a characteristic feature of A/chicken/Guangdong/191/04 (H5N1) HA-triggered JAK/STAT signalling. We therefore examined if targeting JAK3 could block the induction of transcriptional activation of IP-10 and IRF-1 by HA. As shown in **Figure 3A, B**, we demonstrated the ability of the JAK3 inhibitor VI (Calbiochem) to attenuate the phosphorylation of JAK3 while suppressing active NF-κB (**Figure 3A**) in the A549 cells challenged with HA, thus blocking the induction of IP-10 and IRF-1 gene expression (**Figure 3B**). In addition, we also observed significantly lower levels of cytokines/chemokines, including IL-6, IL-8, MCP-1, MIP-1α, MIP-1β and RANTES, in the HA-stimulated A549 cells following treatment with the JAK3 inhibitor VI compared to those with HA stimulation alone (**Figure 3C**).

### Attenuation of the immunopathologic reaction in the Jak3 knockout mice upon HA challenge

**Figure 4:**
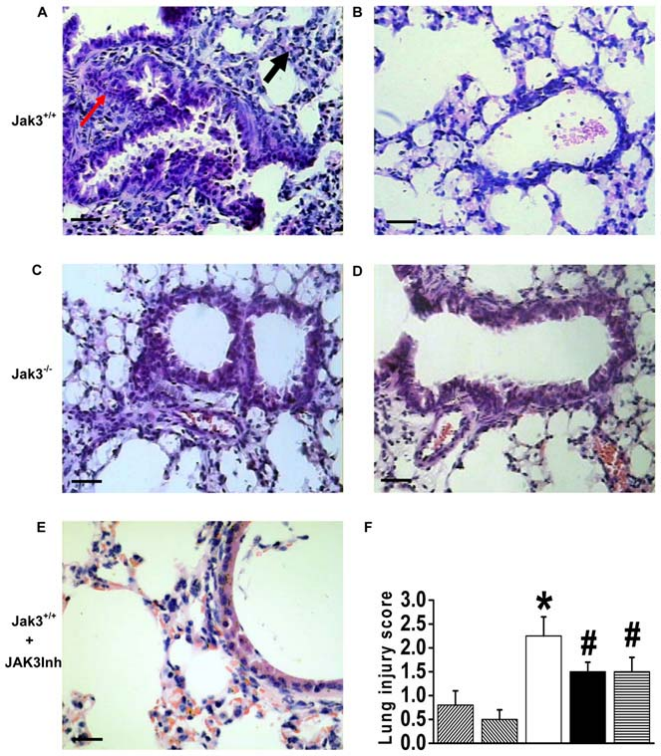
Pathological examination of lung tissues in the Jak3-deficient and wild-type mice following exposure to HA. Jak3^+/+^ and Jak3^−/−^ mice were administered PBS (B, D) or the HA (90 µg per mouse) (A, C) by intratracheal instillation. Meanwhile, the JAK3 inhibitor VI was administered to the Jak3^+/+^ mice prior to the HA instillation (E). Arrows show a marked thickening of the interalveolar septa with infiltration of lymphocytes (black arrow) and interstitial exudation (red arrow). The pathological examination (H&E) of lung tissues was performed at 72 h after HA administration (bar = 50 µm). The lung injury score was assessed in the lung tissues of the mice treated as above (F) (n = 5 per group, 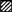, Jak3^+/+^ PBS, 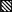, Jak3^−/−^ PBS, 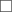, Jak3^+/+^ HA, 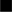, Jak3^−/−^ HA, 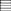, Jak3^+/+^ HA+JAK3Inh). *P<0.05 *vs.* Jak3^+/+^ PBS group; ^#^ *P*<0.05 *vs.* Jak3^+/+^ HA group.

To confirm if the activation of JAK3 enhances HA-driven innate immunity, Jak3 knockout mice were subjected to challenge with an intratracheal instillation of HA. In the lung tissues from HA-challenged Jak3^+/+^ mice, pathologic examination observed diffuse alveolar damage combined with edema, interstitial exudation and hyaline membrane formation; marked thickening of the interalveolar septa; and dense interstitial infiltration by inflammatory cells (**Figure 4A**). However, HA-challenged Jak3^−/−^ mice (**Figure 4C**) or Jak3^+/+^ mice treated with JAK3 inhibitor prior to HA addition (**Figure 4E**) showed a significant decrease in inflammatory cell infiltration with a mild injury score (**Figure 4F**).

**Figure 5:**
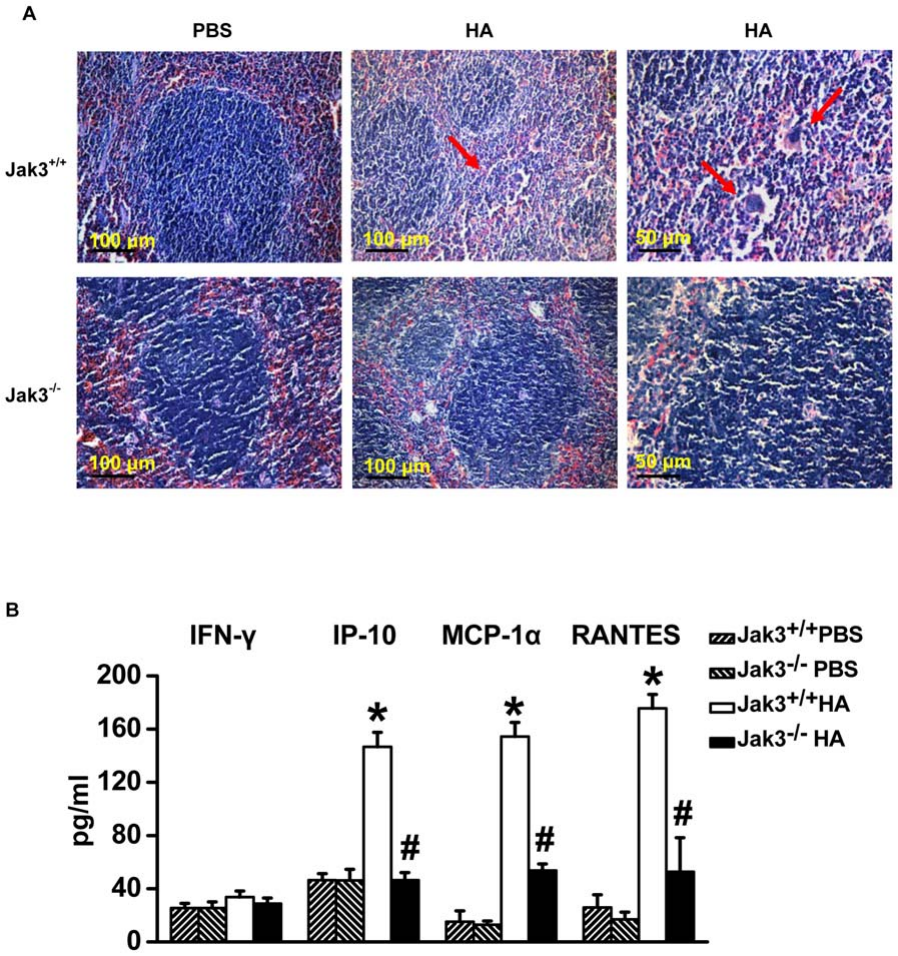
Pathological examination of splenic tissues in the Jak3^−/−^ and Jak3^+/+^ mice following exposure to HA. (A) Haematoxylin/eosin (H&E) staining of paraffin sections of splenic tissues from the Jak3^+/+^ and Jak3^−/−^ mice intratracheally administered with PBS or HA for 72 h. Arrows show the necrosis of lymphocytes. (B) The cytokines/chemokines (IFN-γ, IP-10, MCP-1α and RANTES) that were released from the splenocytes of either Jak3^+/+^ or Jak3^−/−^ mice pretreated with HA or PBS as described above. The measurement of the concentration of the cytokines/chemokines by Liquidchip assay. *P<0.05 *vs.* Jak3^+/+^ PBS group; ^#^ *P*<0.05 *vs.* Jak3^+/+^ HA group.

In addition, we observed that the spleen tissues from the Jak3^+/+^ mice after 72 h of HA intratracheal instillation exhibited swelling, destruction of the local structure of germinal centres and dead lymphocytes (**Figure 5Ab, c**). These changes were not observed in Jak3^−/−^ mice, which appeared normal despite HA treatment (**Figure 5Ae, f**). Compared to the control, the levels of IFN-γ inducible chemokines/cytokines (e.g., IP-10, MCP-1α and RANTES) released from the splenocytes of the HA-pretreated-Jak3^+/+^ mice were significantly increased under basal conditions (*P*<0.05) (**Figure 5B**), but this effect was not seen in the Jak3^−/−^ mice with the HA pretreatment. No significant difference was detected in the expression levels of chemokines in Jak3^−/−^ mice following either PBS or HA instillation (**Figure 5B**). Previous studies have demonstrated that Jak3-dependent cytokine signals were required for the optimal production of IFN-γ in differentiated CD4+ T cells but not for naïve primary CD4+ T cell proliferation and cell cycle regulation *in vitro*, suggesting that these signals promoted the maximal transcription of the IFNγ gene [31]. Taken together, these data indicate that activation of JAK3 signals by HA of H5N1 has a critical role in inducing an intense host response.

### Modulation of a superinflammatory response by inhibition of JAK3-dependent cytokine signals

Given that the JAK3 inhibitor VI has the capability to downregulate NF-κB activation in pulmonary epithelial cells exposed to HA challenge, we tested whether targeting JAK3 signals could prevent superinflammatory responses following bacteria/endotoxin attack. This was tested due to the natural course of the virus, which is highly cytopathic to bronchial and bronchiolar epithelial cells, extending rapidly and diffusely down the respiratory tree and damaging the epithelium sufficiently to breakdown the mucociliary barrier [32].

Both Jak3^+/+^ and Jak3^−/−^ mice were intratracheally inoculated with 90 µg HA or PBS. After 72 h of instillation, the spleen cells were isolated from both groups of mice and cultured in the presence or absence of bacterial endotoxin-LPS at a concentration of 10–50 µg for 12 h and 24 h.

**Figure 6:**
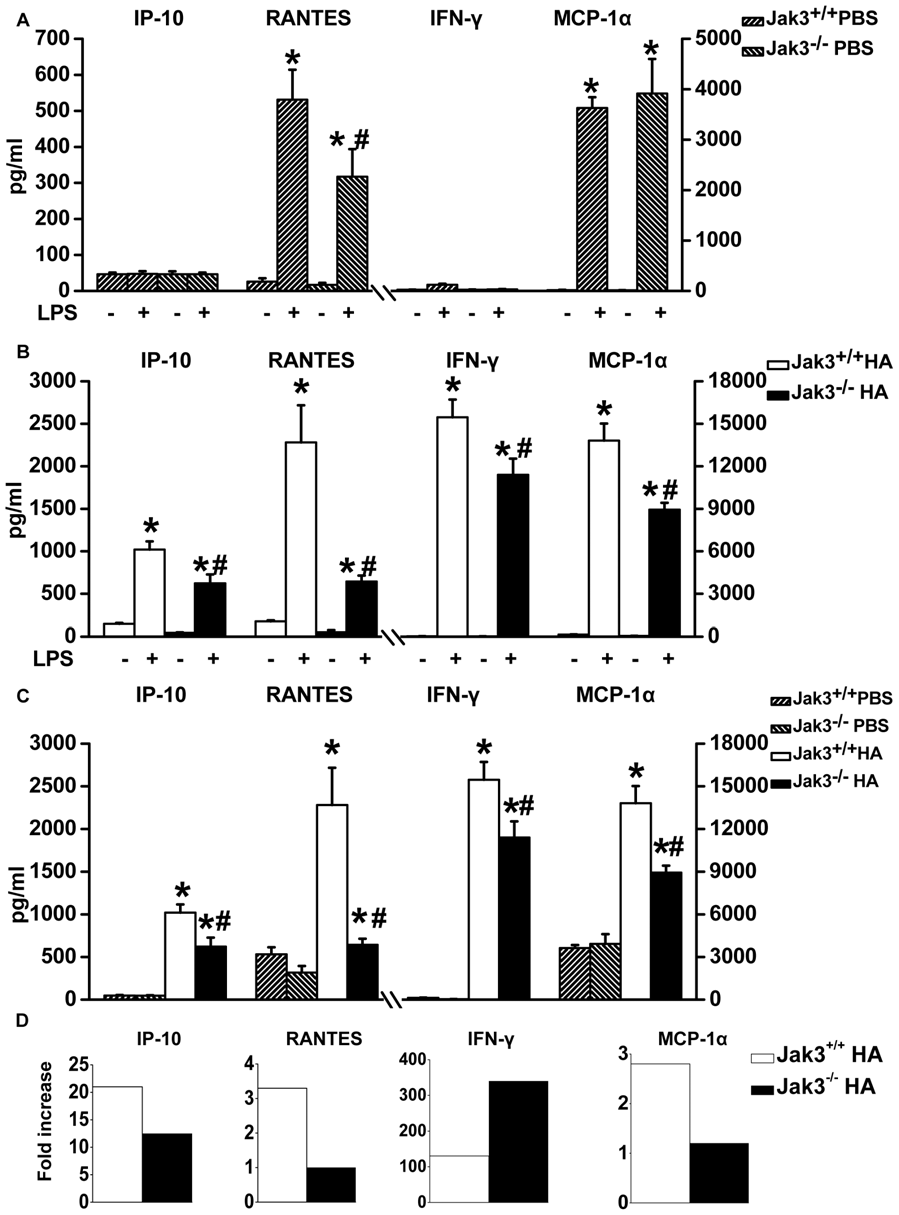
Impact of HA intratracheal instillation on the inflammatory reaction of splenocytes from Jak3^−/−^ mice. Liquidchip assays were performed to determine the cytokines/chemokines (IFN-γ, MCP-1α, IP-10 and RANTES) released from the splenocytes after LPS (20 µg/ml for 12 h) challenge in PBS-pretreated (A) and HA-pretreated (B) mice of either the Jak3^−/−^ or Jak3^+/+^ background. (A) *P<0.05 *vs.* PBS group (Jak3^+/+^ or Jak3^−/−^) without LPS treatment; ^#^ *P*<0.05 *vs.* Jak3^+/+^ PBS group with LPS treatment. (B) *P<0.05 *vs.* HA group (Jak3^+/+^ or Jak3^−/−^) without LPS treatment; ^#^ *P*<0.05 *vs.* Jak3^+/+^ HA group with LPS treatment. A comparison of the levels of the cytokines/chemokines in the supernatants of the splenocytes exposed to LPS from Jak3^−/−^ and Jak3^+/+^ mice with or without HA pretreatment (C). (C) *P<0.05 *vs.* PBS group (Jak3^+/+^ or Jak3^−/−^) with LPS treatment; ^#^ *P*<0.05 *vs.* Jak3^+/+^ HA group with LPS treatment. A comparison of the fold increase of cytokines/chemokines released from the splenocytes following LPS stimulation from Jak3^−/−^ and Jak3^+/+^ mice with HA pretreatment (D).

**Figure 7:**
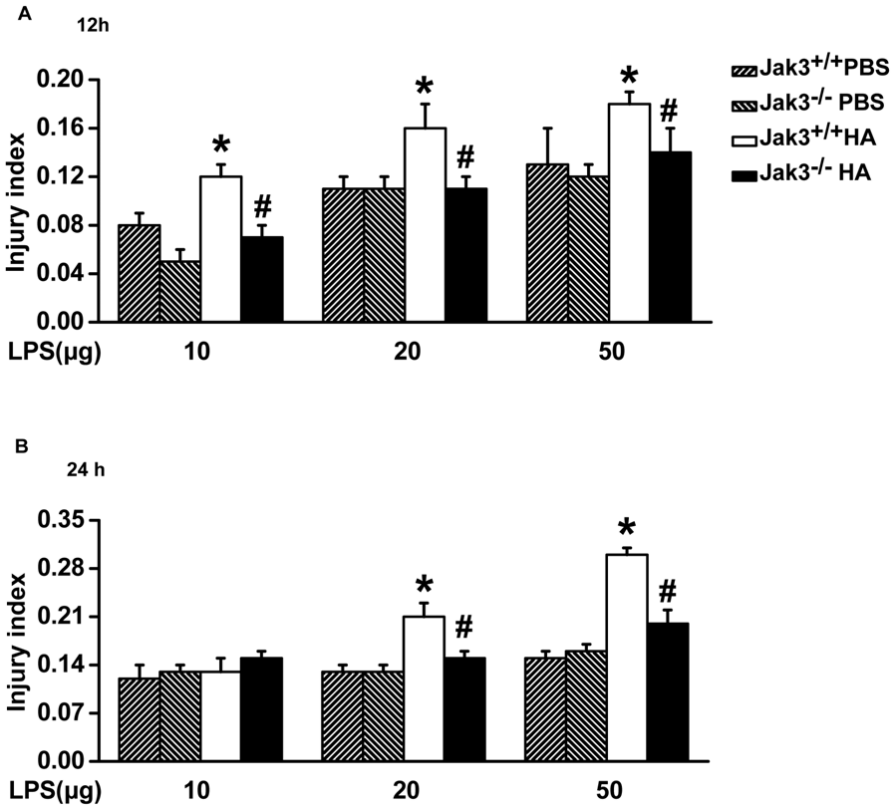
The effects of injury on the splenocytes challenged with LPS in HA-pretreated Jak3^−/−^ mice. The splenocytes isolated from mice pretreated with HA or intratracheal instillation with PBS for 72 h were treated with LPS for 12 h (A) and 24 h (B), and then the cells were subjected to a CCK-8 assay for the evaluation of the injury index. The injury index = (OD value of control cells−OD of LPS-treated cells)/control cells OD. *P<0.05 *vs.* PBS group (Jak3^+/+^ or Jak3^−/−^); ^#^ *P*<0.05 *vs.* Jak3^+/+^ HA group with LPS treatment.

In the splenocytes of Jak3^+/+^ or Jak3^−/−^ mice pretreated with PBS, the stimulation of LPS induced an increase in levels of RANTES and MCP-1α (**Figure 6A**). However, significantly elevated levels of IP-10, RANTES, IFN-γ and MCP-1α were observed in the splenocytes of HA-pretreated mice upon LPS challenge (**Figure 6B**), which were significantly higher in Jak3^+/+^ mice than in Jak3^−/−^ mice, as shown in **Figure 6B**. In response to LPS treatment, the splenocytes from the Jak3^+/+^ mice with HA pretreatment produced higher levels of chemokines/cytokines compared to the PBS-pretreated mice and to the Jak3^−/−^ mice with HA pretreatment (**Figure 6C**). We further made a comparison of the fold increase of the induced chemokines/cytokines between both groups of Jak3^+/+^ and Jak3^−/−^ mice. Except for IFN-γ, there was a much lower fold increase of chemokines/cytokines in the splenocytes with a Jak3 genetic deficiency compared to those with wild-type Jak3 (**Figure 6D**). The injury index of the cultured spleen cells at 12 h (**Figure 7A**) or 24 h (**Figure 7B**) after LPS stimulation was LPS-dose-dependently higher in the Jak3^+/+^ mice than in mice that received PBS pretreatment only or Jak3^−/−^ mice that received the HA pretreatment (*P*<0.05) (**Figure 7**).

These results indicate that sustained JAK3-dependent cytokine signals following virus antigenic challenge predispose the animal to an increased virulence for the subsequent bacterial infection.

## Discussion

Respiratory infection with highly pathogenic influenza A viruses is characterised by the exuberant production of cytokines and chemokines and the enhanced recruitment of innate inflammatory cells. Although alveolar macrophages were originally described as the cell type responsible for pulmonary monocyte recruitment during AIV infection, a recent study by Herold *et al.* suggests instead that the majority of the recruitment results from alveolar epithelial cells that produce high levels of CCL2 (MCP-1), a ligand for CCR2, following infection [33]. This result is in agreement with our findings that show activation of JAK/STAT and NF-κB signalling pathways in pulmonary epithelial cells upon challenge with HA of H5N1 (**Figure 2A**), leading to rapid induction of the IP-10 and IRF-1 genes (**Figure 2B**). In addition, high levels of cytokines/chemokines were produced (**Figure 2C**). These data may represent a mechanism whereby virus antigenic challenge of alveolar epithelial cells constitutes an initiating event for the development of dysregulated innate immunity. It is worthwhile to point out that HA-triggered signalling events are characterised by an unusual process involved in the phosphorylation of JAK3.

The Janus kinases, including JAK1, JAK2, JAK3 and Tyk2, are cytoplasmic protein tyrosine kinases that play an important role in the receptor binding-triggered signal transduction that is mediated through the STAT proteins [34]. The expression patterns of Janus kinase 3 contrast sharply with that of other Janus kinases, which are ubiquitously expressed. JAK3 was found to be more limited in its expression and is found in nature killer (NK) cells and in an NK-like cell line but not in resting T cells or in other tissues [35]. In the present study, we have provided evidence that HA treatment immediately caused phosphorylation of JAK2/3 and STAT1/NF-κB in A549 cells (**Figure 2A**) and mediated the release of cytokines/chemokines (**Figure 2C**), whereas targeting to JAK3 can turn off the signal transduction cascades (**Figure 3**). Our results suggest that JAK3 is inducible upon activation in type II pneumocytes. Following the activation of H5N1 by HA, pulmonary epithelial cells, which actively express JAK3, acquire the capability for the recruitment of inflammatory monocyte-derived DC, NK cells and T cells due to significantly increased release of cytokines/chemokines.

In addition, we show that in Jak3^+/+^ mice, but not in Jak3^−/−^ mice, the HA intratracheal instillation caused acute injury to lungs (**Figure 4**), while necrosis and depletion of lymphocytes were observed in the spleen (**Figure 5A**). However, the splenocytes from HA-pretreated Jak3^+/+^ mice were shown to significantly increase production of IFN-inducible chemokines (e.g., IP10, MCP1) without any stimulation (**Figure 5B**). We have previously demonstrated that the elevation of IP-10 that emerged at the onset of SARS was followed by progressive lymphopenia with a concomitant increase of lactate dehydrogenase, suggesting a depletion of lymphocytes in lymphoid tissues [36]. An early onset of lymphopenia with the pronounced elevation of cytokines/chemokines and apoptotic lymphocytes in the spleen were also observed in patients infected with H5N1 influenza virus, especially those with a severe infection [28]. These data indicate that the early splenic lymphocyte elimination could occur due to an excessive innate immune induction that is dependent on Jak3 signal activation following the antigenic challenge of H5N1, which might contribute to high levels of viral replication.

Activation of NF-κB plays an important role in driving the inflammatory response due to its function as a critical transcriptional activator of proinflammatory cytokines involved in the innate immune response to PAMPs/DAMPs [37]. In the present study, we observed Jak3-dependent signals affecting NF-κB transcriptional activation upon HA stimulation. The splenocytes isolated from the Jak3^−/−^ mice challenged with HA display a resistance against the superinflammatory reaction when they are further exposed to LPS (**Figure 6B,C,D** **and** **Figure 7A,B**), indicating that active expression of JAK3 might be associated with the exacerbation of LPS-mediated NF-κB signalling.

Our present results provide evidence that the inhibition of JAK3 activation enables the negative regulation of NF-κB signalling, further demonstrating that JAK3 is a molecular determinant in the dysregulated innate immune response.

Previous studies using global immune suppressants (such as steroids) have failed to demonstrate protection against lethal influenza virus challenge [38]. It is not surprising that such nonspecific immune suppressants confer no advantage, as a systemic reduction in cell-mediated immunity greatly compromises virus clearance. A recent study demonstrated that combination therapy consisting of an inhibitor of the viral neuraminidase (zanamvir) and two cyclooxygenase 2 (COX2) inhibitors (celecoxib and mesalazine) greatly increased the survival rate of mice infected with a highly pathogenic strain of influenza A/H5N1 virus [39]. Aldridge *et al.* [40] recently reported that prophylactic treatment with the PPAR-γ agonist pioglitazone is sufficient to reduce morbidity and mortality associated with HP influenza A virus infection.

A selective JAK3 inhibitor is considered to be an immunomodulator with extensive application potential because of its specificity without inducing many of the side effects usually caused by corticosteroids. Phase I and II clinical trials proved efficacy and safety of JAK3 inhibition in preventing transplant rejection and eliminating the symptoms of rheumatoid arthritis and psoriasis. CP-690550 which has a similar structure to the JAK3 inhibitor VI [41] used in our study, is currently undergoing Phase III study in patients with active rheumatoid arthritis [42]. Researchers found that CP-690550 strongly inhibited the transcription of RANTES, MIG and IP-10 at 7 days posttransplant [43]. A similar efficiency of the JAK3 inhibitor VI to modulate the proinflammatory cytokines/chemokines was observed *in vitro* and *in vivo* in the present studies (**Figure 3** **and** **4**).

T cell proliferation following activation is an essential aspect for the adaptive immune response against pathogens. When T cells are stimulated under these conditions, their proliferation is γc cytokine independent [44]. Selective inhibition of Jak3-dependent cytokine signals did not affect cell cycle progression following optimal stimulation of T cell receptor plus CD28 [45]. These could explain our findings that a genetic deficiency in Jak3 protected splenic lymphocytes from the intense immune-mediated destruction (**Figure 5**). We presume that patients with severe viral infection may benefit from the treatment with selective JAK3 inhibitors that modulate the dysregulation of cytokine-mediated inflammation but allow T cell proliferation for the adaptive immune response against pathogens.

It is clear that Influenza A viruses (IAV) induces defects in respiratory mucosal immunity that are broad-based and adversely affect the response to a wide range of bacteria. Interactions between the infecting virus and secondary infections due to bacteria that colonise the upper respiratory tract could precipitate the appearance of severe and potentially fatal bacterial pneumonia [32]. Our findings showing the superinflammatory response to LPS of PAMPs in splenocytes from mice pretreated with HA (**Figure 6** **,** **7**) raise the possibility that following viral antigenic challenge, bacteria/endotoxin translocation prolongs and boosts Jak3-dependent cytokine signals, leading to fatal systemic inflammatory response syndrome.

Thus, we suggest that the optimal treatment for the virus-mediated ARDS or/and systemic inflammation may involve combination therapy with efficacy-based antiviral reagents and selective Jak3 inhibitors (e.g., JAK3VI inhibitor or CP-690550).

In summary, we found that challenging pulmonary epithelial cells with the HA from the H5N1 strain of the influenza A virus, resulting in a remarkable activation of the innate immune response via triggering IFN-independent JAK/STAT and NF-κB signal pathways, may be a key mechanism underlying the development of lung damage and lymphocyte apoptosis that avoids immunosurveillance and facilitates efficient viral replication at an early stage of the illness. JAK3 seems to serve as a central signal molecular for the transduction of a ‘super-activated’ immune response to AI-PAMP. We suggest that modulation of the abnormal innate immune inflammation using a selective JAK3 inhibitor could be a novel and valuable strategy for the management of AI-associated severe pneumonia and immune suppression, even though anti-viral therapy is an important first step in recovery.

## Materials and Methods

### Purification of HA protein

Because the baculovirus expression system can produce a high yield of recombinant protein that is usually similar in structure, biological activity and immunological reactivity to the naturally occurring protein, an insect-baculovirus expression system was used for the expression of the recombinant HA protein of AIV H5N1 using the method described by Nwe *et al.* with minor modifications [46]. Using sequence homology, we confirmed that the HA gene of A/chicken/Guangdong/191/04 (H5N1) (GenBank: AY737289) [47] was subcloned into the pFastbacHT plasmid vector, forming a recombinant pFastBacHT-H5HA. Next, pFastBacHT-H5HA was transposited in combination with a baculovirus shuttle vector (bacmid) into MAX Efficiency DH10Bac competent cells by homologous recombination. As predicted, the Bacmid/HA recombinant was identified by PCR amplification of a 4.1 KB DNA fragment (**Figure 1Aa**) following recombination. Using nickel affinity magnet beads, the recombinant HA (64 KDa) of H5N1 was purified from SF9 cells transfected with Bacmid-H5HA and identified by western blotting with an anti-HA (H5N1) antibody (Cat. No. GI-003-006, GeneImmune Inc.), as shown in **Figure 1Ab, c**.

### Mice experiments

B6129S4-Jak3^tm1Ljb^ mice (Jak3^−/−^) and B6129SF2/J mice (Jak3^+/+^) were purchased from Jackson Labs, United States. All mice were housed at a constant temperature (20°C) with a 12-hour light/dark photoperiod and allowed food and water *ad libitum*. The mice were 6 to 8 weeks of age and weighed between 20 and 30 grams. All animal experiments were carried out according to the National Institutes of Health Guide for Care and Use of Laboratory Animals and were approved by the Bioethics Committee of State Key Laboratory of Respiratory Disease, Guangzhou Medical University (Approval ID: 2010-12). Briefly, wild-type or Jak3 knockout mice were randomly divided into two groups (n = 5 in each). After they were anaesthetised with pentobarbital sodium (50 mg/kg), the mice were intratracheally inoculated with 90 µg of HA diluted with 100 µl phosphate-buffered saline (PBS). The control group received an equal volume of sterilised saline without HA. Lung and spleen tissues from the mice were collected 72 h after HA inoculation and fixed in buffered 4% paraformaldehyde (pH 7.4) for histopathological examination.

### Cell culture

A549 cells (A human alveolar epithelial cell line, CCL-185, ATCC, USA) were grown in 75 cm^2^ polystyrene flasks with DMEM (Gibco, USA) supplemented with 10% heat-inactivated foetal bovine serum (FBS) (Gibco, NY, USA). A549 cells were seeded at 1×10^6^ cells per well in 6-well flat-bottom cell culture plates (Corning, NY, USA), which produced a confluent monolayer after overnight incubation at 37°C in a 5% CO_2_ humidified atmosphere. Next, the growth medium was replaced with serum-free DMEM medium and incubated overnight. The cultured A549 cells were either treated with HA or a JAK3 inhibitor VI (760 nM) for 30 min prior to HA addition. Supernatants were collected 12 h after incubation with HA at different concentrations and stored at −70°C until cytokine/chemokine detection.

The spleens were removed from the Jak3^+/+^ and Jak3^−/−^ mice after the mice were intratracheally inoculated with HA for 72 h. The spleens were mechanically disrupted by pressing them through a nylon mesh (pore size, 165 µm) and were deposited in a 25 cm^2^ flask containing 5 ml of RPMI 1640 (Invitrogen Life Technologies). The suspension was passed through a sterile nylon mesh (pore size, 50 µm) to obtain the splenocytes. After the lysis of erythrocytes by treatment with Tris/NH_4_Cl buffer, the pooled splenocytes were suspended with complete tissue culture medium consisting of RPMI 1640 supplemented with 10% of heat-inactivated FBS (Invitrogen Life Technologies), 100 U/ml penicillin and streptomycin [48].

### Western blot analysis

A549 cells were lysed in RIPA buffer [50 mM Tris (pH 7.5), 150 mM NaCl, 1% NP-40, 0.1% SDS, 1 mM EDTA, 1 mM NaN_3_, 1 mM PMSF, 2 µg/ml aprotinin, 2 µg/ml leupeptin]. Lysates were cleared by centrifugation, and supernatants were stored in aliquots at −80°C until further use. The protein was quantified using a BCA assay kit (Pierce, USA), and 100 µg was used for SDS-PAGE electrophoresis. After the proteins were transferred from the gel onto a polyvinylidene fluoride membrane (DuPont-New England Nuclear, Boston, MA), the membrane was blocked with 5% non-fat dried milk in Tris-buffered saline and Tween-20 for 1 h, followed by further incubation of the membrane with 5% non-fat dried milk containing the primary antibody at 4°C overnight. Immunodetection of target proteins was performed with primary antibodies for total or phosphorylated JAK1, JAK2, JAK3, STAT1 and NF-κB (Cell Signalling, Frankfurt, Germany). After washing, the secondary antibody [goat-anti-rabbit IgG conjugated to HRP (Cell Signalling, Frankfurt, Germany)] was added and incubated for an additional 1 h. Immunoreactive bands were developed using an ECL chemiluminescent substrate (Pierce, Rockford, USA), and digital scanning was performed in an Image Station 2000 (Kodak, US). For all experiments, GAPDH (Novus Biologicals, USA) was detected simultaneously to confirm equal protein loading.

### RT-PCR

After treatment with HA or vehicle for the indicated period, A549 cells were harvested, and total RNA was isolated using TriZol Reagent (Invitrogen). Then, the reverse transcription reaction was conducted using SuperScript™ III reverse transcription reagents (Invitrogen). We amplified previously generated cDNA by PCR using the following specific primers for IP-10, IRF-1 and GAPDH: for IP-10, forward 5′- AGGAACCTCCAGTCTCAGCA -3′ and reverse 5′- GGCAGTGGAAGTCCATGAAG -3′; for IRF-1, forward 5′- CTTAAGAACCCGGCAACCTCTGCCTTC -3′ and reverse 5′- GATATCTGGCAGGGAGTTCATG-3′; and for GAPDH, forward 5′- GGTGAAGGTCGGAGTCAACG -3′ and reverse 5′-CAAAGTTGTCATGGATGACC-3′, with product sizes of 757 bp, 405 bp and 497 bp, respectively. All primers were purchased from Invitrogen (California, USA). The PCR amplification was performed using a Biometra T-GRADIENT thermal cycler (Nordic BioSite, Täby, Sweden) using the following protocol: reactions were predenatured at 94°C for 120 s, denatured at 94°C for 30 s, then cycled at 55°C for 50 s and 72°C for 60 s for 30 cycles. PCR amplicons were analysed on 1.5% agarose gels, stained with ethidium bromide, and subsequently visualised. To confirm use of equal amounts of RNA in each experiment, all samples were assessed for GAPDH mRNA expression.

### Luminex assay

The quantification of multiple cytokines/chemokines was performed using the Luminex assay LiquidChip system (Panomics, CA, USA), which is a bead-based system for immunoassays that allows for the simultaneous assaying of multiple analytes in a single sample [49]. The cytokines/chemokines included IL-2, IL-4, IL-6, IL-8, tumour necrosis factor α (TNF-α), IFN-γ, IP-10, MCP-1, macrophage inflammatory protein 1 alpha (MIP-1α), MIP-1β and regulated upon activation normal T cell expressed and secreted (RANTES). Supernatants of the HA-treated A549 cells were analysed on a LiquidChip system according to the manufacturer's instructions. The isolated splenocytes from the Jak3^+/+^ and Jak3^−/−^ mice with or without HA pretreatment were subjected to culture in the absence or presence of lipopolysaccharide (LPS, 20 µg/ml). After 12 h or 24 h of culture, the supernatants of the splenocytes were collected for the LiquidChip assay.

### Lung and spleen histology

For analysis by light microscopy, lung and spleen tissues were fixed with freshly prepared 4% paraformaldehyde in PBS (pH 7.4) for 36 h and embedded in paraffin. Tissue sections (5 µm) were stained with haematoxylin and eosin (H&E) to enable the histological evaluation of lung and spleen tissues. Two investigators blinded to the group assignments analysed the samples and determined the level of lung injury according to the semiquantitative scoring outlined below. All lung fields were examined for each sample at ×20 magnification. The assessment of histological lung injury was performed as follows: 0, normal; 1, <25% the lung section exhibits interstitial congestion and inflammatory cell infiltration; 2, 25–50% the lung section exhibits interstitial congestion and inflammatory cell infiltration; 3, 50–75% the lung section exhibits consolidation and inflammatory cell infiltration. The mean score was used for the comparison between groups.

### Lymphocyte injury assay

The isolated spleen cells (1×10^6^ per well) were seeded into 96-well tissue culture plates and stimulated with LPS at different concentrations (10, 20 or 50 µg) or PBS for 12 or 24 h at 37°C and 5% CO_2_; 2-[2-methoxy-4-nitrophenyl]-3-[4-nitrophenyl]-5-[2,4-disulfophenyl]-2H-tetrazolium monosodium salt (WST-8, Cell-Counting Kit-8® (Dojindo Molecular Technologies, Inc., Gaithersburg, MD) 10 µl per well) was added as described previously [50]. The plates were incubated for 4 h, and the optical density (OD) at 450 nm was measured using a microplate reader (BioRad, Hercules, CA). Each sample was analysed in three replicates, and the injury index was calculated using the following formula: injury index = (mean OD of control group–LPS-stimulated group)/(mean OD of control group).

### Statistical analysis

Comparisons among treatment groups were performed with a one-way-ANOVA test. A *P* value less than 0.05 was considered statistically significant.

## References

[1] Swayne DE, Suarez DL (2000). Highly pathogenic avian influenza.. Rev Sci Tech.

[2] WHO (2010).

[3] Peiris JS, de Jong MD, Guan Y (2007). Avian influenza virus (H5N1): a threat to human health.. Clin Microbiol Rev.

[4] Gu J, Xie Z, Gao Z, Liu J, Korteweg C (2007). H5N1 infection of the respiratory tract and beyond: a molecular pathology study.. Lancet.

[5] Zhang Z, Zhang J, Huang K, Li KS, Yuen KY (2009). Systemic infection of avian influenza A virus H5N1 subtype in humans.. Hum Pathol.

[6] de Jong MD, Simmons CP, Thanh TT, Hien VM, Smith GJ (2006). Fatal outcome of human influenza A (H5N1) is associated with high viral load and hypercytokinemia.. Nat Med.

[7] Mer M, Richards GA (1998). Corticosteroids in life-threatening varicella pneumonia.. Chest.

[8] Gomersall CD, Joynt GM, Lam P, Li T, Yap F (2004). Short-term outcome of critically ill patients with severe acute respiratory syndrome.. Intensive Care Med.

[9] Jorens PG, Van Damme J, De Backer W, Bossaert L, De Jongh RF (1992). Interleukin 8 (IL-8) in the bronchoalveolar lavage fluid from patients with the adult respiratory distress syndrome (ARDS) and patients at risk for ARDS.. Cytokine.

[10] Beigel JH, Farrar J, Han AM, Hayden FG, Hyer R (2005). Avian influenza A (H5N1) infection in humans.. N Engl J Med.

[11] Maines TR, Szretter KJ, Perrone L, Belser JA, Bright RA (2008). Pathogenesis of emerging avian influenza viruses in mammals and the host innate immune response.. Immunol Rev.

[12] Baskin CR, Bielefeldt-Ohmann H, Tumpey TM, Sabourin PJ, Long JP (2009). Early and sustained innate immune response defines pathology and death in nonhuman primates infected by highly pathogenic influenza virus.. Proc Natl Acad Sci U S A.

[13] Uiprasertkul M, Kitphati R, Puthavathana P, Kriwong R, Kongchanagul A (2007). Apoptosis and pathogenesis of avian influenza A (H5N1) virus in humans.. Emerg Infect Dis.

[14] Cheung CY, Poon LL, Lau AS, Luk W, Lau YL (2002). Induction of proinflammatory cytokines in human macrophages by influenza A (H5N1) viruses: a mechanism for the unusual severity of human disease?. Lancet.

[15] To KF, Chan PK, Chan KF, Lee WK, Lam WY (2001). Pathology of fatal human infection associated with avian influenza A H5N1 virus.. J Med Virol.

[16] Chan MC, Cheung CY, Chui WH, Tsao SW, Nicholls JM (2005). Proinflammatory cytokine responses induced by influenza A (H5N1) viruses in primary human alveolar and bronchial epithelial cells.. Respir Res.

[17] Opitz B, van Laak V, Eitel J, Suttorp N (2010). Innate immune recognition in infectious and noninfectious diseases of the lung.. Am J Respir Crit Care Med.

[18] Beutler BA (2009). TLRs and innate immunity.. Blood.

[19] O'Neill LA (2008). ‘Fine tuning’ TLR signaling.. Nat Immunol.

[20] van Riel D, Munster VJ, de Wit E, Rimmelzwaan GF, Fouchier RA (2006). H5N1 Virus Attachment to Lower Respiratory Tract.. Science.

[21] van Riel D, Munster VJ, de Wit E, Rimmelzwaan GF, Fouchier RA (2007). Human and avian influenza viruses target different cells in the lower respiratory tract of humans and other mammals.. Am J Pathol.

[22] Hiemstra PS, Bals R (2004). Series introduction: Innate host defense of the respiratory epithelium.. J Leukoc Biol.

[23] Knowles MR, Boucher RC (2002). Mucus clearance as a primary innate defense mechanism for mammalian airways.. J Clin Invest.

[24] Kagnoff MF, Eckmann L (1997). Epithelial cells as sensors for microbial infection.. J Clin Invest.

[25] Sandrock C, Kelly T (2007). Clinical review: update of avian influenza A infections in humans.. Crit Care.

[26] Steinberg KP, Hudson LD, Goodman RB, Hough CL, Lanken PN (2006). Efficacy and safety of corticosteroids for persistent acute respiratory distress syndrome.. N Engl J Med.

[27] Annane D, Sebille V, Bellissant E (2006). Effect of low doses of corticosteroids in septic shock patients with or without early acute respiratory distress syndrome.. Crit Care Med.

[28] Gambotto A, Barratt-Boyes SM, de Jong MD, Neumann G, Kawaoka Y (2008). Human infection with highly pathogenic H5N1 influenza virus.. Lancet.

[29] Jaruga B, Hong F, Kim WH, Gao B (2004). IFN-gamma/STAT1 acts as a proinflammatory signal in T cell-mediated hepatitis via induction of multiple chemokines and adhesion molecules: a critical role of IRF-1.. Am J Physiol Gastrointest Liver Physiol.

[30] Schroder K, Hertzog PJ, Ravasi T, Hume DA (2004). Interferon-gamma: an overview of signals, mechanisms and functions.. J Leukoc Biol.

[31] Shi M, Lin TH, Appell KC, Berg LJ (2008). Janus-kinase-3-dependent signals induce chromatin remodeling at the Ifng locus during T helper 1 cell differentiation.. Immunity.

[32] Morens DM, Taubenberger JK, Fauci AS (2008). Predominant role of bacterial pneumonia as a cause of death in pandemic influenza: implications for pandemic influenza preparedness.. J Infect Dis.

[33] Herold S, von Wulffen W, Steinmueller M, Pleschka S, Kuziel WA (2006). Alveolar epithelial cells direct monocyte transepithelial migration upon influenza virus infection: impact of chemokines and adhesion molecules.. J Immunol.

[34] Cetkovic-Cvrlje M, Tibbles HE (2004). Therapeutic potential of Janus kinase 3 (JAK3) inhibitors.. Curr Pharm Des.

[35] Kawamura M, McVicar DW, Johnston JA, Blake TB, Chen YQ (1994). Molecular cloning of L-JAK, a Janus family protein-tyrosine kinase expressed in natural killer cells and activated leukocytes.. Proc Natl Acad Sci U S A.

[36] Jiang Y, Xu J, Zhou C, Wu Z, Zhong S (2005). Characterization of cytokine/chemokine profiles of severe acute respiratory syndrome.. Am J Respir Crit Care Med.

[37] Hatada EN, Krappmann D, Scheidereit C (2000). NF-kappaB and the innate immune response.. Curr Opin Immunol.

[38] Salomon R, Hoffmann E, Webster RG (2007). Inhibition of the cytokine response does not protect against lethal H5N1 influenza infection.. Proc Natl Acad Sci U S A.

[39] Zheng BJ, Chan KW, Lin YP, Zhao GY, Chan C (2008). Delayed antiviral plus immunomodulator treatment still reduces mortality in mice infected by high inoculum of influenza A/H5N1 virus.. Proc Natl Acad Sci U S A.

[40] Aldridge JR, Moseley CE, Boltz DA, Negovetich NJ, Reynolds C (2009). TNF/iNOS-producing dendritic cells are the necessary evil of lethal influenza virus infection.. Proc Natl Acad Sci U S A.

[41] Adams C, Aldous DJ, Amendola S, Bamborough P, Bright C (2003). Mapping the kinase domain of Janus Kinase 3.. Bioorg Med Chem Lett.

[42] Changelian PS, Flanagan ME, Ball DJ, Kent CR, Magnuson KS (2003). Prevention of organ allograft rejection by a specific Janus kinase 3 inhibitor.. Science.

[43] West K (2009). CP-690550, a JAK3 inhibitor as an immunosuppressant for the treatment of rheumatoid arthritis, transplant rejection, psoriasis and other immune-mediated disorders.. Curr Opin Investig Drugs.

[44] Lantz O, Grandjean I, Matzinger P, Di Santo JP (2000). Gamma chain required for naive CD4+ T cell survival but not for antigen proliferation.. Nat Immunol.

[45] Hacker G, Bauer A, Villunger A (2006). Apoptosis in activated T cells: what are the triggers, and what the signal transducers?. Cell Cycle.

[46] Nwe N, He Q, Damrongwatanapokin S, Du Q, Manopo I (2006). Expression of hemagglutinin protein from the avian influenza virus H5N1 in a baculovirus/insect cell system significantly enhanced by suspension culture.. BMC Microbiol.

[47] Wan XF, Ren T, Luo KJ, Liao M, Zhang GH (2005). Genetic characterization of H5N1 avian influenza viruses isolated in southern China during the 2003-04 avian influenza outbreaks.. Arch Virol.

[48] Lewandowski D, Marquis M, Aumont F, Lussier-Morin AC, Raymond M (2006). Altered CD4+ T cell phenotype and function determine the susceptibility to mucosal candidiasis in transgenic mice expressing HIV-1.. J Immunol.

[49] Hutchinson KL, Villinger F, Miranda ME, Ksiazek TG, Peters CJ (2001). Multiplex analysis of cytokines in the blood of cynomolgus macaques naturally infected with Ebola virus (Reston serotype).. J Med Virol.

[50] Miyamoto T, Min W, Lillehoj HS (2002). Lymphocyte proliferation response during Eimeria tenella infection assessed by a new, reliable, nonradioactive colorimetric assay.. Avian Dis.

